# A new deep learning algorithm of 12-lead electrocardiogram for identifying atrial fibrillation during sinus rhythm

**DOI:** 10.1038/s41598-021-92172-5

**Published:** 2021-06-17

**Authors:** Yong-Soo Baek, Sang-Chul Lee, Wonik Choi, Dae-Hyeok Kim

**Affiliations:** 1 Division of Cardiology, Department of Internal Medicine, Inha University College of Medicine and Inha University Hospital, 27 Inhang-ro, Jung-gu, Incheon, 22332 Republic of Korea; 2 Department of Computing Engineering, Inha University, 100 Inha-ro, Incheon, 22212 Republic of Korea; 3 Department of Information and Communication Engineering, Inha University, 100 Inha-ro, Michuhol-gu, Incheon, 22212 Republic of Korea; 4 DeepCardio Inc., Incheon, Republic of Korea

**Keywords:** Cardiology, Health care

## Abstract

Atrial fibrillation (AF) is the most prevalent arrhythmia and is associated with increased morbidity and mortality. Its early detection is challenging because of the low detection yield of conventional methods. We aimed to develop a deep learning-based algorithm to identify AF during normal sinus rhythm (NSR) using 12-lead electrocardiogram (ECG) findings. We developed a new deep neural network to detect subtle differences in paroxysmal AF (PAF) during NSR using digital data from standard 12-lead ECGs. Raw digital data of 2,412 12-lead ECGs were analyzed. The artificial intelligence (AI) model showed that the optimal interval to detect subtle changes in PAF was within 0.24 s before the QRS complex in the 12-lead ECG. We allocated the enrolled ECGs to the training, internal validation, and testing datasets in a 7:1:2 ratio. Regarding AF identification, the AI-based algorithm showed the following values in the internal and external validation datasets: area under the receiver operating characteristic curve, 0.79 and 0.75; recall, 82% and 77%; specificity, 78% and 72%; F1 score, 75% and 74%; and overall accuracy, 72.8% and 71.2%, respectively. The deep learning-based algorithm using 12-lead ECG demonstrated high accuracy for detecting AF during NSR.

## Introduction

Atrial fibrillation (AF) is one of the most important public health problems and a significant cause of increasing health care costs worldwide^1^. AF is the most common form of arrhythmia and is reported to increase mortality and the risk of ischemic stroke, heart failure, and dementia in patients^2,3^. AF is confirmed based on 12-lead electrocardiogram (ECG) findings; however, it is difficult to identify AF, especially paroxysmal AF (PAF), from ECGs acquired during normal sinus rhythm (NSR) because of low detection by conventional methods and the silent nature of PAF^4^. Conventional methods, such as Holter ECG monitoring and event recorder examination, rely on the detection of symptoms over a relatively short period. ECG patches, such as smartwatches, have recently shown a diagnostic AF yield of 34%^5^. It has been reported that ECG monitoring with an implantable loop recorder (ILR) was superior to conventional follow-up for detecting AF after cryptogenic stroke^3,6^. However, smartwatches and ILRs are not widely available because of their cost and invasiveness, making them less accessible to some patients and doctors. These methods also have insurance issues on a case-by-case basis. Therefore, a new cost-effective strategy to meet the “unmet need” and improve AF detection is needed in the future. Meanwhile, the progression of AF can cause electrical and structural changes, manifesting as subtle changes on normal ECGs^7,8^. However, even for cardiologists, it is impossible to distinguish the NSR of a patient with PAF from that of a healthy person without AF on an ECG. A recent report showed good performance of artificial intelligence (AI) using a convolutional neural network for point-of-care identification of AF using ECGs acquired during NSR in patients with PAF^9^. We hypothesized that we could identify the subtle ECG changes present in a standard 12-lead ECG during NSR in patients with PAF using a deep learning algorithm. To evaluate this hypothesis, we trained, validated, and tested a recurrent neural network (RNN) deep learning algorithm using NSR ECGs in PAF and healthy individuals in a tertiary hospital.

## Results

### Patient characteristics

**Figure 1:**
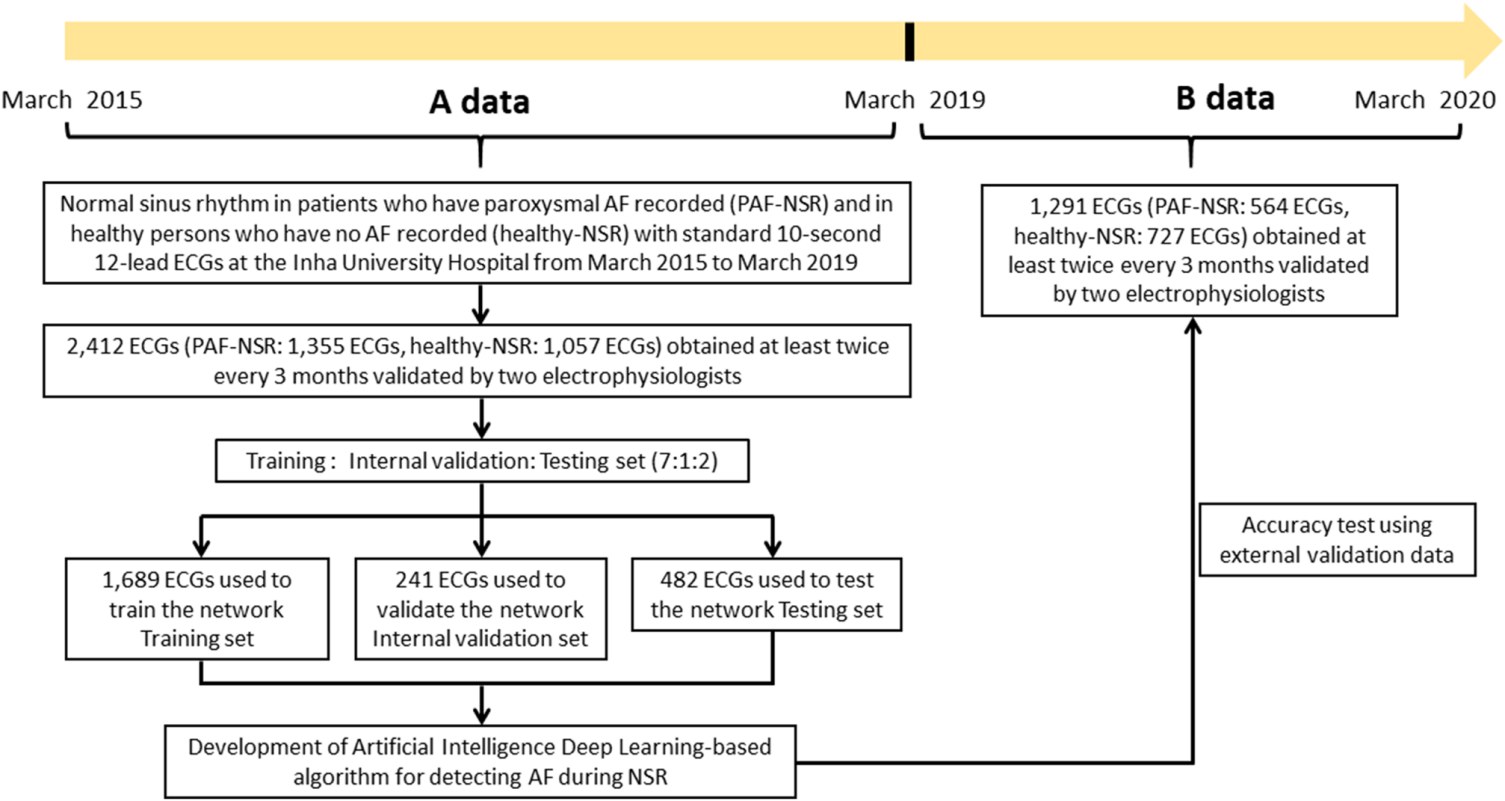
Patient flow diagram showing the selection of the study population and the creation of the study datasets. ECGs were allocated to the training, internal validation, and testing datasets in a 7:1:2 ratio to assure a robust and reliable dataset. *PAF* paroxysmal atrial fibrillation; *ECG* electrocardiogram.

The baseline characteristics and comorbidities of the development and external validation datasets are shown in Table 1. The mean age of the participants was 61.2 ± 12.8 years. The mean body mass index was 24.1 ± 3.9 kg/m^2^. The mean CHA_2_DS_2_-VASc score of NSR in patients who have PAF recorded (PAF-NSR group) was 2.8 ± 1.9. Patients with PAF-NSR in both data A and B had higher HR and prolonged PR interval, QRS duration, and corrected QT interval than healthy persons who have no AF recorded (Table 1). We included 2412 NSR ECGs for analysis (NSR in healthy persons who have no AF recorded (healthy-NSR): 1057 ECGs; PAF-NSR: 1355 ECGs); especially, 1677 ECGs from 426 patients, 238 ECGs from 60 patients, and 497 ECGs from 124 patients were used in the training, validation, and testing datasets, respectively. We performed external validation using dataset B, which included 1291 12-lead ECGs (healthy-NSR: 727; PAF-NSR: 564) (Fig. 1).

**Table 1:** Patient characteristics and electrocardiographic findings at enrollment.

	Dataset A				Dataset B				
	Overall	PAF-NSR	Healthy-NSR	*P-value	Overall	PAF-NSR	Healthy-NSR	*P-value	†*P*-value
	(n = 2,412)	(n = 1,355)	(n = 1,057)		(n = 1,291)	(n = 564)	(n = 727)		
Age, years	61.2 ± 12.8	66.5 ± 13.0	54.7 ± 9.2	< 0.001	59.6 ± 13.2	65.2 ± 12.6	55.3 ± 11.9	< 0.001	< 0.001
Female sex, n (%)	1,544 (65.0)	571 (43.3)	973 (92.1)	< 0.001	813 (63.0)	206 (36.5)	607 (83.5)	< 0.001	0.234
Body mass index, kg/m^2^	24.1 ± 3.9	24.2 ± 4.3	23.9 ± 3.2	0.345	24.4 ± 3.4	25.7 ± 4.2	23.7 ± 2.7	< 0.001	0.275
Hypertension, n (%)	1,021 (42.3)	837 (63.5)	184 (17.4)	< 0.001	613 (47.5)	463 (82.1)	150 (20.6)	< 0.001	0.009
Diabetes mellitus, n (%)	387 (16.0)	304 (23.0)	83 (7.9)	< 0.001	341 (26.4)	273 (48.4)	68 (9.4)	< 0.001	< 0.001
Heart failure, n (%)	239 (9.9)	239 (18.1)	0 (0)	< 0.001	149 (11.5)	145 (25.7)	4 (0.6)	< 0.001	0.177
Stroke, n (%)	78 (3.2)	74 (5.6)	4 (0.4)	< 0.001	54 (4.2)	52 (9.2)	2 (0.3)	< 0.001	0.165
TIA, n (%)	50 (2.1)	41 (3.1)	9 (0.9)	< 0.001	22 (1.7)	16 (2.8)	6 (0.8)	< 0.001	0.456
Vascular disease, n (%)	369 (15.2)	2 (1.3)	0 (0)	0.500	142 (11.0)	131 (23.2)	11 (1.5)	< 0.001	< 0.001
CHA_2_DS_2_-VASc score	2.2 ± 1.7	2.8 ± 1.9	1.4 ± 0.9	< 0.001	1.9 ± 1.3	2.5 ± 1.4	1.4 ± 1.0	< 0.001	< 0.001
**ECG findings**									
Heart rate, bpm	67.9 ± 11.6	68.6 ± 12.9	67.3 ± 10.1	0.003	67.9 ± 10.5	68.6 ± 11.5	67.4 ± 9.6	0.038	0.938
PR interval, msec	162.2 ± 19.4	164.0 ± 19.6	160.4 ± 19.0	< 0.001	164.8 ± 19.3	167.0 ± 20.1	163.1 ± 18.4	< 0.001	< 0.001
QRS duration, msec	90.4 ± 9.5	91.8 ± 9.8	89.0 ± 8.9	< 0.001	91.0 ± 9.4	91.8 ± 9.5	90.5 ± 9.3	0.013	0.046
QT interval, msec	405.8 ± 33.5	407.6 ± 37.6	403.9 ± 28.6	0.003	400.6 ± 30.2	402.8 ± 33.9	398.8 ± 26.7	0.02	< 0.001
QTc, msec	427.6 ± 26.1	430.9 ± 30.2	424.2 ± 20.6	< 0.001	422.6 ± 23.3	426.5 ± 25.0	419.6 ± 21.4	< 0.001	< 0.001
Duration between an AF episode and PAF-NSR ECG, months	NA	3.8 ± 4.7	NA	NA	NA	3.3 ± 4.5	NA	NA	0.112

Values are expressed as n (%) or as means ± standard deviations. NA means not applicable.

*P value of Student’s t-test or chi-square test between PAF-normal and healthy-NSR participants.

^†^*P* value of Student’s t-test or chi-square test between datasets A and B.

bpm: beats per minute; CHA2DS2-VASc: a score taking into account congestive heart failure, hypertension, age ≥ 75 years, diabetes mellitus, previous stroke/transient ischemic attack, vascular disease, age 65–74 years, and sex (female); *ECG* electrocardiography; *PAF* paroxysmal atrial fibrillation; *QRSd* QRS duration; *TIA* transient ischemic attack.

### Optimal section for AF detection during NSR in ECG

We hypothesized that the vicinity of the P-wave before the QRS complex would be important for differentiating AF during NSR. We conducted an experiment, which consisted of gradually increasing the section size including the P-wave section starting from the onset of the QRS complex. By incrementing 10 samples within the range of 20–180 samples, the experiment was performed by designating it as a sequence length to observe the trend of classification accuracy.

As shown in the Supplemental Figure, we found that the optimal interval to detect subtle changes of AF detection during a sinus rhythm was approximately within 240 ms (about 120 sample size) before the QRS complex by the validation accuracy test. The moving average is computed by averaging the validation accuracy values within a range of [S − 20, S + 20] for the sample size S tested in [70, 180], which is equivalent to the period of 80 ms.

### Performance of the model for identifying AF

**Figure 2:**
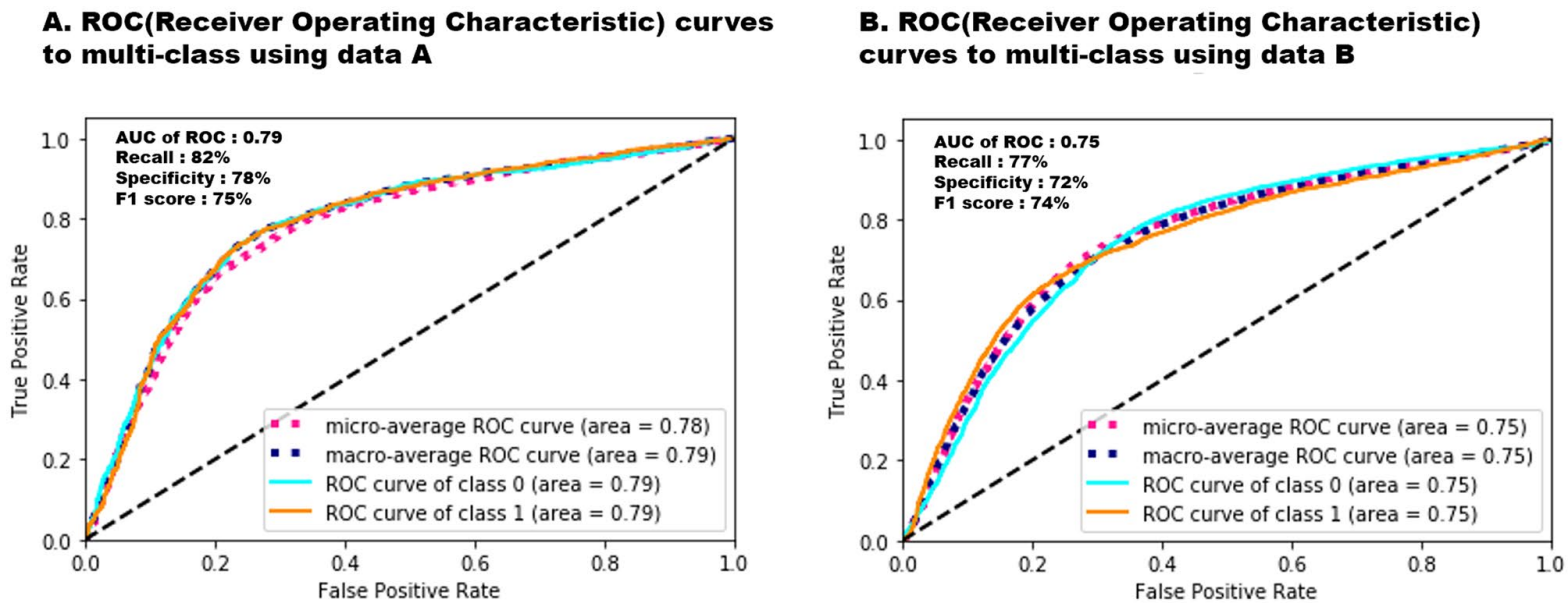
Multiclass ROC curves with deep neural networks applied in the internal and external datasets. The micro-average and macro-average AUC derived from the ROC curve of the AI algorithm is calculated during the internal and external validations (0.78 [95% CI 0.76–0.80], 0.79 [95% CI 0.78–0.80] for internal validation dataset; 0.75 [95% CI 0.74–0.76], 0.75 [95% CI 0.74–0.76] for external validation dataset). *ROC* receiver operating characteristic; *AUC* area under the ROC curve. Class 0: healthy-NSR, Class 1: PAF-NSR.

The suggested model produced an F1 score of 75% (95% confidence interval [CI] 73.0–76.9), recall of 82.0% (80.3–83.6) in the PAF-NSR group, a specificity of 78% (76.1–79.8) in the healthy-NSR group, and an overall accuracy of 73% (71.6–74.3; Table 2). As shown in Fig. 2, during external validation, the algorithm showed an area under the receiver operating characteristic (ROC) curve (AUC) of 0.75 (0.74–0.76), a recall of 77% (75.1–80.2), a specificity of 72% (69.8–73.8), an F1 score of 74% (71.0–76.1), and an overall accuracy of 71.2% (69.8–73.5) for identifying AF.

**Table 2:** AI model performance.

	Precision	Recall (sensitivity)	F1-score*	Support^a^
Healthy-NSR	0.78 (0.76–0.80)	0.64 (0.62–0.66)	0.70 (0.68–0.72)	2,096
PAF-NSR	0.69 (0.67–0.71)	0.82 (0.80–0.84)	0.75 (0.73–0.77)	2,074
Accuracy			0.73 (0.72–0.74)	4,170
Macro avg	0.74 (0.73–0.75)	0.73 (0.72–0.74)	0.73 (0.72–0.74)	4,671
Weighted avg	0.74 (0.73–0.75)	0.73 (0.72–0.74)	0.73 (0.72–0.74)	4,671

Data in parentheses represent the 95% confidence intervals. *AI* artificial intelligence; *avg* average; *PAF* paroxysmal atrial fibrillation.

*F1 Score (balanced F-score) is the harmonic mean of precision and recall, and calculated as follows: F1 score = 2 (precision × recall) / (precision + recall).

^a^Support is the number of occurrences of each class when it is true.

### Application of ECG interpretation using deep learning analysis

**Figure 3:**
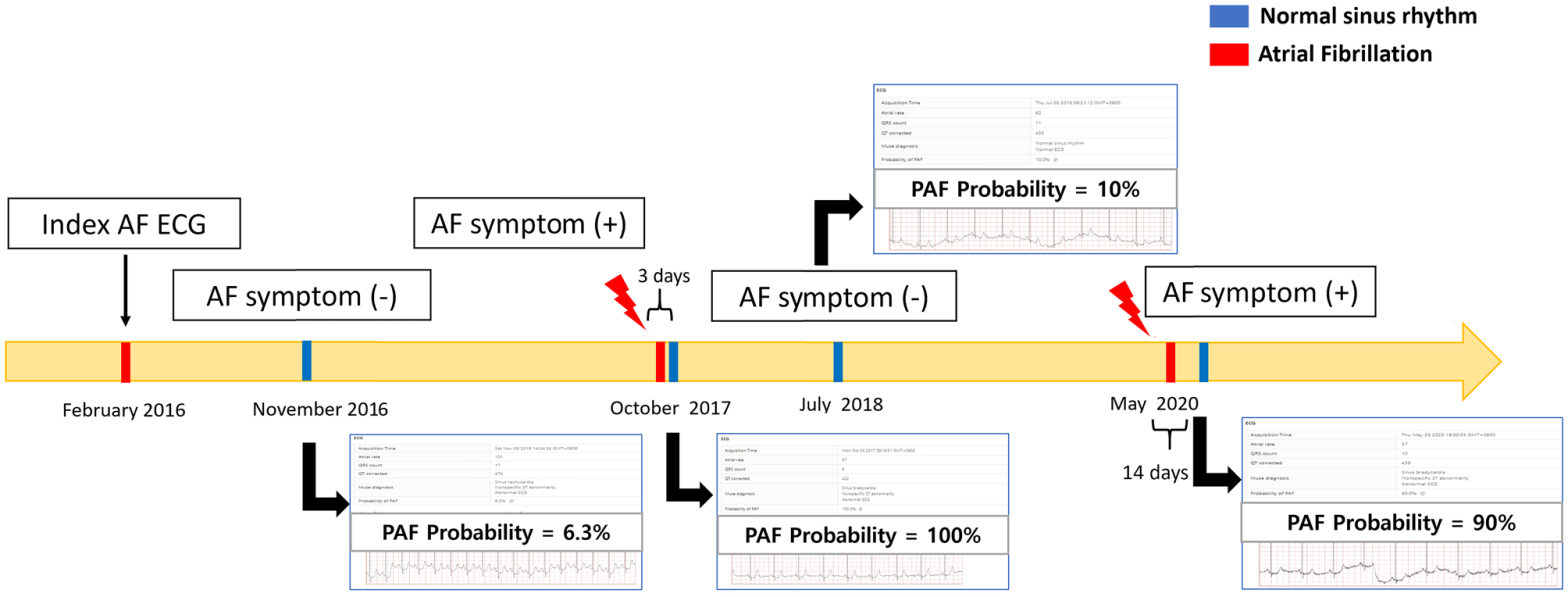
The serial changes of the probability of PAF using AI deep learning algorithm program according to acquired ECG dates. A 72-year-old man diagnosed with PAF is calculated to have AF with probabilities of 90% and 100% by the AI program during normal sinus rhythm, observed after his AF episode had terminated (red lightning bolt). Moreover, he was calculated to have AF with probabilities of 6.3% and 10% when there was no AF symptom. AF symptom is defined as palpitations, fatigue, dizziness, dyspnea, chest pain, and anxiety during AF. *AI* artificial intelligence; *ECG* electrocardiogram; *NSR* normal sinus rhythm; *PAF* paroxysmal atrial fibrillation.

We developed an RNN-based AI application that can be used for analyses in real-time on computers in our hospital after internal validation of the RNN-based deep learning algorithm. Using our application, there were interesting findings revealed by the NSR ECGs. For instance, when taken on a date close to the date of documented AF or when an AF symptom was present, it tended to have high detection probability, and low AF detection probability was noted in the absence of AF symptoms when multiple serial ECGs were assessed from the same patient. As shown in Fig. 3, the probability of PAF using a deep learning algorithm program could change according to the dates of ECG acquisition. For example, a 72-year-old man diagnosed with PAF was calculated to have AF with probabilities of 90% and 100% by the AI program during NSR, acquired after his AF episode had terminated, and to have AF with probabilities of 6.3% and 10% in the absence of AF symptoms.

## Discussion

We analyzed the predictive value and the optimal section in an ECG for identifying AF during NSR using a deep learning algorithm. The AI-deep learning algorithm developed to estimate the probability of PAF during NSR using a 12-lead ECG was excellent for identifying PAF (recall of 82%, specificity of 78%, F1 score of 75%, and overall accuracy of 72.8%). The suggested model showed a reliable harmonic mean of precision and recall (F1 score) for identifying PAF during NSR compared with the models used in recently published studies^9,10^. The model showed that the optimal interval to detect subtle changes of PAF was within 0.24 s before the QRS complex in a 12-lead ECG.

Deep learning models usually require access to large and accurate datasets^11^. Despite the relatively small size of our data compared to those in the previous studies, our model showed favorable recall and accuracy^9,10^. This could be attributed to the use of accurate ECG data for training and validation of deep learning verified by two electrophysiologists and the detection of optimal intervals for AF detection. It has been reported that P-wave analysis calculated on a standard surface ECG could be used to identify patients with PAF^12–14^. We intended to recognize the subtle but significant differences among PAF-NSR and healthy-NSR ECGs carefully through this approach despite the relatively small data size. It is expected that through the use of this model, the amount of data required for a diagnosis would reduce greatly, making it easy to apply to actual clinical trials.

Opportunistic screening for AF in patients aged ≥ 65 years during other examinations, such as blood pressure checks, has detected AF in approximately 1.4% of patients^15^. The detection rates of AF using repeated snapshot handheld ECG devices and continuous recordings, such as patches or ILRs, were 1–2.5% per day (3.8% per week) and 22–34% per year, respectively^16,17^. However, these monitoring devices are invasive and expensive^18^. Although it is difficult to perform a head-to-head comparison among these various modalities for AF detection because of different techniques used and heterogeneity of patients enrolled, AI using ECG could have a good performance to detect patients with PAF using a single 12-lead ECG, which is a rapid, simple, and inexpensive point-of-care test. Currently, there is an unmet need for a method to increase AF detection with good sensitivity. Our algorithm showed excellent performance for recall of identifying AF. ECG and Holter monitoring are short-term monitoring methods that usually show NSR in one or more tests, even in patients with AF. However, patients’ preferences for intensive long-term monitoring pose limitations for AF detection. Therefore, the use of AI to increase the accuracy of AF diagnosis would be very useful in pre-screening, as it would save unnecessary inspection time and cost. With continuous ECG monitoring over extended periods for people aged 65 years and older, one-fourth to one-third of them would have brief AF episodes. The use of our model in this population could be a cost-effective alternative for AF detection.

Data from a Swedish registry helped identify two major gaps in AF-related stroke prevention, representing 33% of all ischemic strokes^19^. AF was not detected before the stroke in 9% of all stroke cases^15^. In these patients, AF screening and stroke prevention, such as appropriate anticoagulation prescription, would be needed for the prevention of recurrent strokes. Pre-screening for AF using AI could be helpful in reducing the evidence-practice gap of oral anticoagulant (OAC) prescriptions in these populations.

The cost-effectiveness is likely to be a result of earlier diagnosis of AF and initiation of treatment to reduce stroke risk, as stroke is a severe event with a high economic burden^20^. Since stroke is a serious event with a large economic burden, cost effectiveness is most likely the result of early diagnosis of AF and initiation of treatment to reduce the risk of stroke. Early rhythm-control therapy was associated with a lower risk of adverse cardiovascular outcomes compared with usual care in patients diagnosed early with AF according to findings from the EAST-AFNET 4 trial^21^. Accurate early diagnosis and proper clinical management of AF are expected to contribute to improving patient and population health outcomes by ensuring that patients receive appropriate treatment. We expect that if AI performance becomes more accurate in the near future, it will play a role in this first step of AF screening.

It has been demonstrated that the maintenance of AF provokes ion channel changes and leads to a marked shortening of the atrial effective refractory (AER) period, a reversion of its physiological rate adaptation, and an increase in rate, inducibility, and stability of AF; all these changes were completely reversible within 1 week of sinus rhythm^22^. AF is a progressive disease associated with progressive electrical and structural remodeling and a gradual increase in AF burden^23^. Delayed recurrence of AF after AF ablation might be related to AF progression^24^. The time-course of AER involves a transitional period associated with the progression and maintenance of AF^25^. AF progression shows patient-specific patterns of the atrial activation rate^26^. Our model showed differences in acquired AF probabilities of NSR ECG according to AF episodes in the same patients (Fig. 3). It suggested that the duration between an AF episode and the length of PAF-normal ECG recording might be associated with subtle changes in ECG, indicating AF progression. Additionally, our study showed that AI could identify this subtle difference even in NSR. This could explain the reflected reversible electrical remodeling when there were AF symptoms or episodes. In this study, patients with PAF-NSR had higher HR and prolonged PR interval, QRS duration, and corrected QT interval than healthy persons who have no AF recorded. Previous studies showed that several ECG changes had been identified in patients with AF, including prolonged PR interval, P wave duration, and QT interval, and left ventricular hypertrophy^23,26,27^. Although we cannot understand and interpret these ECG changes because of a so-called “black box” limitation of a deep learning algorithm in terms of the approach to the decision for detecting AF, we have assumed that these changes related to atrial remodeling might have influenced our deep learning decision process.

We have evaluated an AI-based deep learning algorithm for the identification of AF during NSR. Further studies should evaluate the hypothesis that combining ECG analysis with AI and clinical comorbidities could enhance AF prediction. Such algorithms could be useful stratification tools for patients at risk for developing new-onset AF, especially in those with cryptogenic stroke. While there have been many reported traditional predictors of PAF after cryptogenic stroke, only a few studies focused on ECG analysis using AI^9,10,28^. Attia and colleagues reported that AI could help in identifying the point of care of individuals with AF by using 649,931 ECGs acquired during NSR^9^. It has been reported that deep learning network could help in identifying new-onset AF and AF-related stoke using the 1.6 million 12-lead ECGs by Raghunath and colleagues^10^. In other words, the intensive analysis of ECG provided by the deep neural network might detect subtle and multifaceted perturbations of ECG and to identify AF-related stroke patients predicted to be at a high risk of AF. Although further research is needed, these deep learning algorithms may be able to identify a high-risk subset of patients with potential stroke who may benefit from empirical anticoagulant therapy. Improved risk stratification would allow more patient-centered intervention and patient-tailored decision making for better AF management. Increasing awareness and detection of undiagnosed AF and administering OAC for thromboprophylaxis remains an ongoing issue. The increased detection yield of AF by AI could lead to the establishment of effective thromboprophylaxis with OACs to overcome the treatment gap between aspirin and OACs^29^. In the future, efforts should be directed at the primary prevention of AF to provide the basis for fine-tuning patient-tailored decision making. Furthermore, it could be used to predict responsibility of AF treatment, such as electrical and pharmacological cardioversions, and AF catheter ablation.

Several limitations in this study should be considered. First, as this study was a retrospective research conducted in one tertiary hospital in Korea, it is necessary to validate the model with patients from other hospitals and countries. Study enrollment duration of data B is different from that of data A because of the limitation of a retrospective study. Baseline characteristics and electrocardiographic findings were different between data A and B. However, patients with PAF-NSR in both data A and B had similar characteristics and pattern of electrocardiographic findings. A prospective study is warranted to establish its usefulness in AF patients as a new feasible and non-invasive screening tool. The interpretation of deep learning models is challenging, and benefiting from deep learning models requires access to large datasets. Therefore, future studies to improve the interpretability of the developed deep learning models and to identify the right size of the training and test datasets are warranted. Second, despite focusing on a specific area on the ECG, the accurate rationale behind AI decision making remains unknown because of the nature of AI; therefore, this needs to be further explored. Recently, explainable AI has been studied, and the automatic detection of bias and the ability to explain its decision making process could be made possible in the near future^30^. Furthermore, we focused on developing screening tools for AF based on 12-lead ECGs. Despite the favorable performance of our deep learning algorithm, overcoming false positives and negatives to identify the optimal treatment and predict prognosis remains an important issue. Nonetheless, health professionals can be alerted to potential occurrences of AF in the population with a higher risk of AF by the suggested algorithm, and additional evaluation with ECG monitoring might be warranted. Although it is difficult to rely on AI as a direct factor in clinical decisions involving the administration of drugs, such as novel OACs or antiarrhythmics, the algorithm can predict AF with high sensitivity to improve AF detection, which is an unmet need in the field, especially for patients with cryptogenic stroke.

In conclusion, the deep learning-based algorithm using 12-lead ECGs may discriminate “hidden” AF during NSR. Further studies are needed to evaluate their possible use in future prognostic models for precise decision making in daily practice.

## Methods

### Study design and population

This retrospective cohort study included adult participants (age ≥ 18 years) with standard 12-lead ECGs acquired at least twice every 3 months to ensure accuracy of PAF and health-NSR group classification of AF rhythms recorded at the Inha University Hospital. ECG XML raw data to access and extract for AI use in our hospitals have been stored since 2015. Dataset A, acquired from March 2015 to March 2019, was used for development and internal validation, and dataset B, acquired from April 2019 to April 2020 after AI development, was used for external validation. All ECGs were acquired at a sampling rate of 500 Hz using a GE-Marquette ECG machine (Marquette Tools, Milwaukee, WI, USA), with the raw data stored as XML documents using the MUSE data management system in relational databases. We defined PAF as episodes of AF lasting < 48 h, which terminated spontaneously within 7 days or terminated following electric or pharmacological cardioversion within 48 h^14^. The first recorded ECG with AF was defined as the index ECG; subsequent NSR ECGs were defined as PAF-NSR ECGs. We identified healthy-NSR ECGs as the NSR ECGs of healthy individuals on the health screening list at our hospital, with NSR ECGs acquired at least twice every 3 months to ensure accuracy of health-NSR group classification of no AF rhythms recorded. Patients who continued to use antiarrhythmic drugs for > 3 months were excluded to rule out antiarrhythmic effects. Two electrophysiologists reviewed all the ECGs with corrections made to the diagnostic labels as necessary. Figure 1 shows the dataset creation and analysis strategy, which was devised to ensure a robust and reliable dataset for training, validating, and testing the network. The study protocol was approved by the Institutional Review Board of the Inha University Hospital (2018-630 and 2019-10-038) and complied with the principles of the Declaration of Helsinki. The need for obtaining patients’ informed consent was waived owing to impracticality and minimal risk of harm.

### Development of the AI algorithm for identifying AF during NSR

The AI algorithm was developed using an RNN to manage sequential data reflecting the ECG characteristics^31,32^. An ECG is a graphical display of the heart’s electrical activity depicting changes in voltage over time through electrodes. These electrodes detect subtle electrical changes resulting from cardiac muscle depolarization and repolarization during each cardiac cycle. Changes in the normal ECG pattern occur in numerous heart abnormalities, including arrhythmia. We have chosen the RNN of deep neural networks, which have advantages in dealing with time-series data, such as ECG data^33^. The bi-directional connection is added so that time flow can be considered in forward and backward passes, and long short-term memory is used to maintain a series of information in the short and long terms. We extracted and analyzed XML data from the MUSE data management system to minimize artifacts. All data files were stored in the XML format on a GE MAC5500 machine (GE Healthcare, Chicago, IL, USA). The ECGs were originally measured on 12 leads, but because of the deviceʼs data storage method, only data from eight leads were stored, excluding XMLʼs Lead III, aVR, aVL, and aVF. The data from these four leads can be calculated with simple arithmetic expressions, and it is common practice to approximate the data with these operations. Therefore, in this study, only the eight measured signals of leads I, II, V1, V2, V3, V4, V5, and V6 were used. The signals were measured for 10 s on each lead simultaneously. When the Base 64 encoded value was read, eight one-dimensional arrays for each XML file were obtained. As a 10-s signal has multiple pulses and the heart rate varies from person to person, approximately 10 or more pulses can be obtained per person. For training, we used all the individual beats sampled from each recording after discarding a few highly noisy signals. The validation and test were performed by grouping the beats from a single recording. The result was finally computed by the ratio of test detected beats as PAF-NSR to the total number of beats in the recording. We separated the training, validation, and test sets to include a group of patient recordings in only one set.

AF has characterized atrial fibrillatory waves and an irregular ventricular rate on 12-lead ECG. We hypothesized and verified that the vicinity of the P-wave before the QRS complex would be important for differentiating AF during NSR. To verify this hypothesis, we tested the accuracy of the binary classification by assessing whether each case was a PAF-NSR or healthy-NSR ECG; then, the data were evaluated once with five-fold cross-validation. The results demonstrated that the accuracy of PAF detection starts to improve when approximately ≥ 100 samples are used in the test. The experiment for the optimal sample size to identify AF was performed at the specific range of the R-R interval that was significantly related to PAF ^12,34^. We reweighted the input ECG signal f(t) using the window function g(t). For the optimal interval to detect subtle changes in PAF-NSR, we used the rectangular function as follows:

$$ g\left( t \right) = \left\{ {\begin{array}{*{20}l}    1 \hfill & {{\text{if}}~\;140~\; \le t <  - 20} \hfill  \\     0 \hfill & {otherwise} \hfill  \\   \end{array} } \right. $$

**Figure 4:**
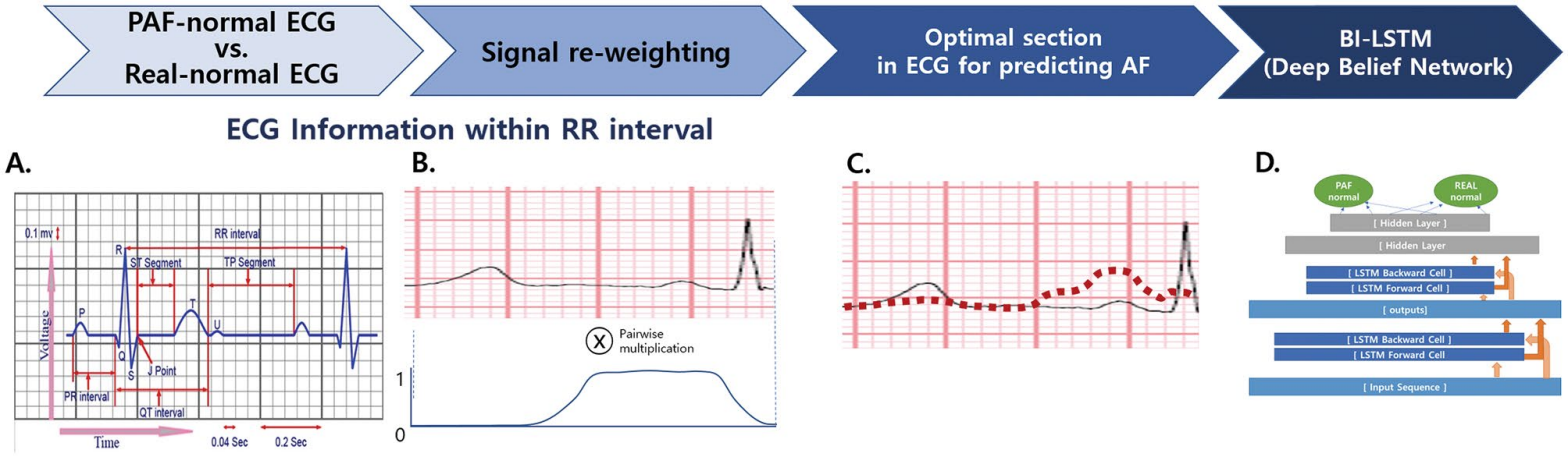
Optimal section for AF detection during NSR. (**a**) The experiment for the optimal sample size to identify AF was performed at a certain range of the R–R interval, where we reweighted the input EEG signal f(t) using the window function g(t) (**b**). The reweighted signal h(t) is computed by the equation h(t) = f(t) × g(t) and illustrated by the red dotted curve (**c**). This process clarifies the value ranges that particularly affect the trained model along the time-axis. The bi-directional connection is added so that the time flow can be considered in forward and backward passes, and long short-term memory is used to maintain a series of information in the short and long terms (**d**). *NSR* normal sinus rhythm, *AF* paroxysmal atrial fibrillation; *LSTM* long short-term memory.

The reweighted signal h(t) was computed as follows: h(t) = f(t) × g(t). This process clarified the value ranges that particularly affect the trained model along the time-axis. The bi-directional connection was added so that time flow could be considered in forward and backward passes, and long short-term memory was used to maintain a series of information in the short and long terms (Fig. 4).

**Figure 5:**
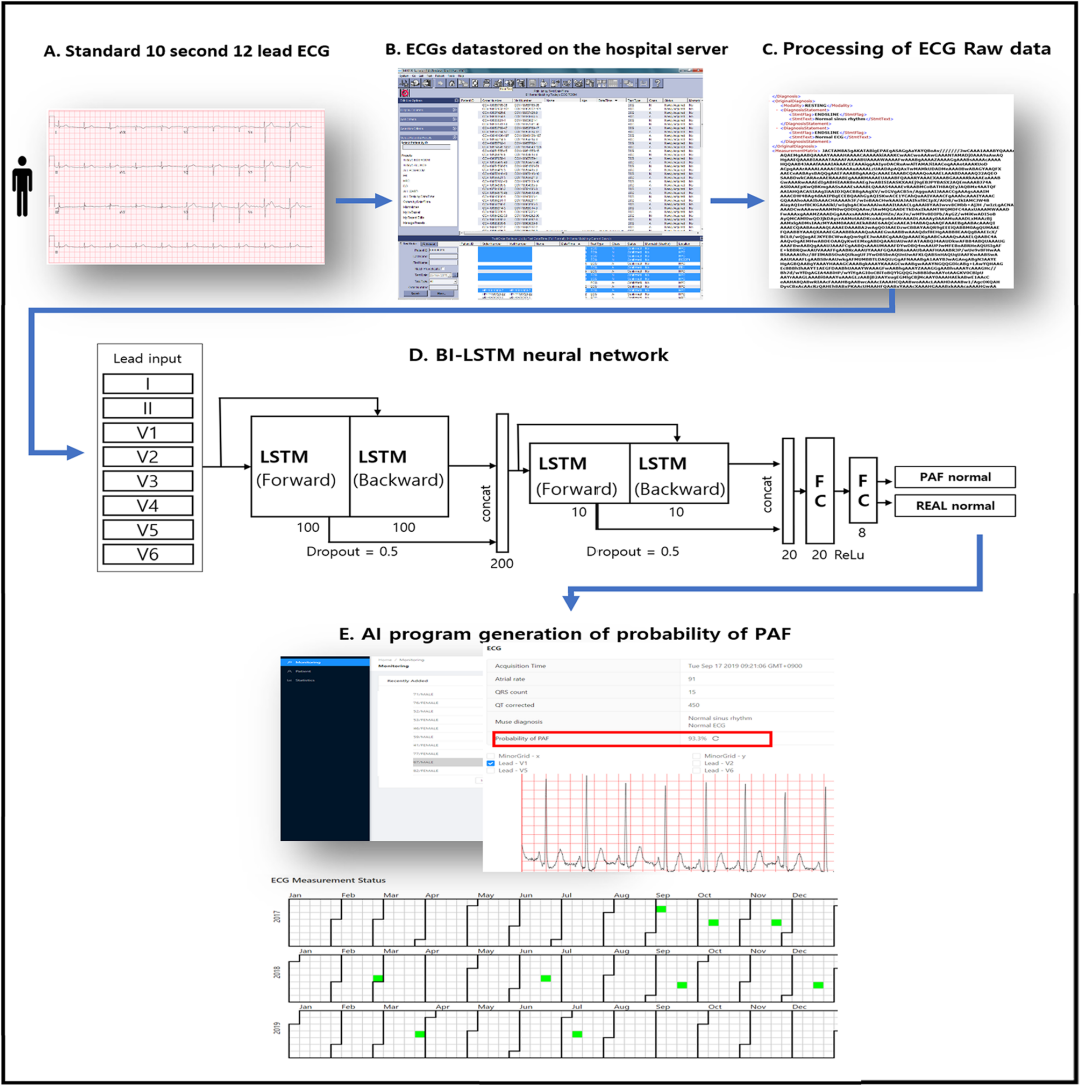
Description of the artificial intelligence algorithm for detecting PAF. All raw ECG data were stored as XML documents using the MUSE data management system in a relational database server. PAF probability is calculated through our developed AI algorithm using an RNN with two-dimensional convolution (red box). *AI* artificial intelligence; *ECG* electrocardiogram; *LSTM* long short-term memory; *PAF* paroxysmal atrial fibrillation; *RNN* recurrent neural network.

A ROC curve was created to test and validate the datasets and assess the AUC of the AI-enabled ECGs acquired during NSR to determine whether AF was present. Using the ROC curve for the small internal validation set, the probability threshold was set and applied to the testing dataset to derive the accuracy, sensitivity, specificity, and F1 score of the testing dataset. After the internal validation of this RNN-based deep learning algorithm, we developed AI applications that can be used on computers in our hospital. Continuous ECG data since 2019 were gathered and analyzed in real-time. Figure 5 describes the schematic representation of the AI algorithm and its application for detecting PAF.

### Statistical analysis

Continuous variables are reported as means ± standard deviations or medians and interquartile ranges, and categorical variables are expressed as percentages and frequencies. Comparisons between the two groups were performed using the independent sample t-test or chi-square test. The performance of the AI model was measured using the AUC and ROC curves for predicting dataset accuracy, recall (sensitivity), specificity, and F1 score. The recall is the ratio of correctly predicted positive observations to all observations. F1 score (balanced F-score) is the harmonic mean of precision and recall. A two-sided value of *P* ≤ 0.05 was considered statistically significant. Statistical analyses were performed using SPSS statistical software (SPSS version 21.0 for Windows, SPSS Inc., Armonk, NY, USA).

## Supplementary Information

Supplementary Legend.

Supplementary Information 1.

## Data Availability

The data collected from the Inha University Hospital during this study are patient data obtained under the institutional review boards’ ethical approval. The corresponding author agrees to share de-identified individual participant data, the study protocol, and the statistical analysis plan with academic researchers following completion of a data use agreement specifying that this information cannot be shared. The coding used to train the AI model is dependent on annotation, infrastructure, and hardware and therefore, cannot be released.

## References

[1] Kim D (2018). 10-year nationwide trends of the incidence, prevalence, and adverse outcomes of non-valvular atrial fibrillation nationwide health insurance data covering the entire Korean population. Am. Heart J..

[2] Gladstone DJ (2014). Atrial fibrillation in patients with cryptogenic stroke. N. Engl. J. Med..

[3] Sanna T (2014). Cryptogenic stroke and underlying atrial fibrillation. N. Engl. J. Med..

[4] Freedman B, Schnabel R, Calkins H (2019). Opportunistic electrocardiogram screening for atrial fibrillation to prevent stroke. JAMA Cardiol..

[5] Perez MV (2019). Large-scale assessment of a smartwatch to identify atrial fibrillation. N. Engl. J. Med..

[6] Thijs VN (2016). Predictors for atrial fibrillation detection after cryptogenic stroke. Neurology.

[7] Nattel S, Harada M (2014). Atrial remodeling and atrial fibrillation: recent advances and translational perspectives. J. Am. Coll. Cardiol..

[8] Van Gelder IC, Hemels MEW (2006). The progressive nature of atrial fibrillation: a rationale for early restoration and maintenance of sinus rhythm. Europace.

[9] Attia ZI (2019). An artificial intelligence-enabled ECG algorithm for the identification of patients with atrial fibrillation during sinus rhythm: a retrospective analysis of outcome prediction. Lancet.

[10] Raghunath S (2021). Deep neural networks can predict new-onset atrial fibrillation from the 12-lead ECG and help identify those at risk of atrial fibrillation–related stroke. Circulation.

[11] Abadi, M. et al. Deep learning with differential privacy. *Proceedings of the 2016 ACM SIGSAC Conference on Computer and Communications Security*, 308–318 (2016).

[12] Dilaveris PE, Gialafos JE (2001). P-wave dispersion: a novel predictor of paroxysmal atrial fibrillation. Ann. Noninvasive Electrocardiol..

[13] Steinberg JS (1993). Value of the P-wave signal-averaged ECG for predicting atrial fibrillation after cardiac surgery. Circulation.

[14] Hindricks G (2021). 2020 ESC guidelines for the diagnosis and management of atrial fibrillation developed in collaboration with the European Association for Cardio-Thoracic Surgery (EACTS). Eur Heart J.

[15] Freedman B (2017). Screening for atrial fibrillation: a report of the AF-SCREEN international collaboration. Circulation.

[16] Goldberger JJ, Mitrani RD (2018). Electrocardiographic monitoring for prevention of atrial fibrillation-associated cardioembolic stroke. JAMA.

[17] Maheshwari A (2019). Refining prediction of atrial fibrillation-related stroke using the P2-CHA2DS2-VASc score. Circulation.

[18] Force UPST (2018). Screening for atrial fibrillation with electrocardiography: US preventive services task force recommendation statement. JAMA.

[19] Freedman B (2018). Major progress in anticoagulant uptake for atrial fibrillation at last: does it translate into stroke prevention?. Eur. Heart J..

[20] Xu XM (2018). The economic burden of stroke care in England, Wales and Northern Ireland: Using a national stroke register to estimate and report patient-level health economic outcomes in stroke. Eur. Stroke J..

[21] Kirchhof P (2020). Early rhythm-control therapy in patients with atrial fibrillation. N. Engl. J. Med..

[22] Wijffels MC, Kirchhof CJ, Dorland R, Allessie MA (1995). Atrial fibrillation begets atrial fibrillation. A study in awake chronically instrumented goats. Circulation.

[23] Heijman J, Voigt N, Nattel S, Dobrev D (2014). Cellular and molecular electrophysiology of atrial fibrillation initiation, maintenance, and progression. Circ. Res..

[24] Baek Y-S (2016). Delayed recurrence of atrial fibrillation 2years after catheter ablation is associated with metabolic syndrome. Int J Cardiol.

[25] Martins RP (2014). Dominant frequency increase rate predicts transition from paroxysmal to long-term persistent atrial fibrillation. Circulation.

[26] Lillo-Castellano JM (2020). Personalized monitoring of electrical remodelling during atrial fibrillation progression via remote transmissions from implantable devices. Europace.

[27] German DM, Kabir MM, Dewland TA, Henrikson CA, Tereshchenko LG (2016). Atrial fibrillation predictors: importance of the electrocardiogram. Ann. Noninvasive Electrocardiol..

[28] Thijs VN (2016). Predictors for atrial fibrillation detection after cryptogenic stroke: results from CRYSTAL AF. Neurology.

[29] Lowres N, Giskes K, Hespe C, Freedman B (2019). Reducing stroke risk in atrial fibrillation: adherence to guidelines has improved, but patient persistence with anticoagulant therapy remains suboptimal. Korean Circ. J..

[30] Wen Z, Hou B, Jiao L (2017). Discriminative nonlinear analysis operator learning: when cosparse model meets image classification. IEEE Trans. Image Process..

[31] Chung, J., Gulcehre, C., Cho, K. & Bengio, Y. Empirical evaluation of gated recurrent neural networks on sequence modeling. arXiv:1412.3555 (2014).

[32] Bahdanau, D., Cho, K. & Bengio, Y. Neural machine translation by jointly learning to align and translate. arXiv:1409.0473 (2014).

[33] Goto S, Goto S (2019). Application of neural networks to 12-lead electrocardiography- current status and future directions. Circ. Rep..

[34] Aytemir K (2000). P wave dispersion on 12-lead electrocardiography in patients with paroxysmal atrial fibrillation. Pacing Clin. Electrophysiol..

