# Supplementary material for: A new deep learning algorithm of 12-lead electrocardiogram for identifying atrial fibrillation during sinus rhythm
Source: Sci Rep. 2021 Jun 17;11:12818. doi: 10.1038/s41598-021-92172-5 (PMC8211689; doi:10.1038/s41598-021-92172-5)
Supplement: Supplementary file 1 — Supplementary Legend. [file 41598_2021_92172_MOESM1_ESM.docx]

**Supplemental Figure.** The validation accuracy graph by experiment for the optimal sample size to predict AF

Red line, validation accuracy; blue dotted line, moving average; 1 sample size is approximately 2 msec
