# Supplementary figures and images for: A new deep learning algorithm of 12-lead electrocardiogram for identifying atrial fibrillation during sinus rhythm
Source: Sci Rep. 2021 Jun 17;11:12818. doi: 10.1038/s41598-021-92172-5 (PMC8211689; doi:10.1038/s41598-021-92172-5)

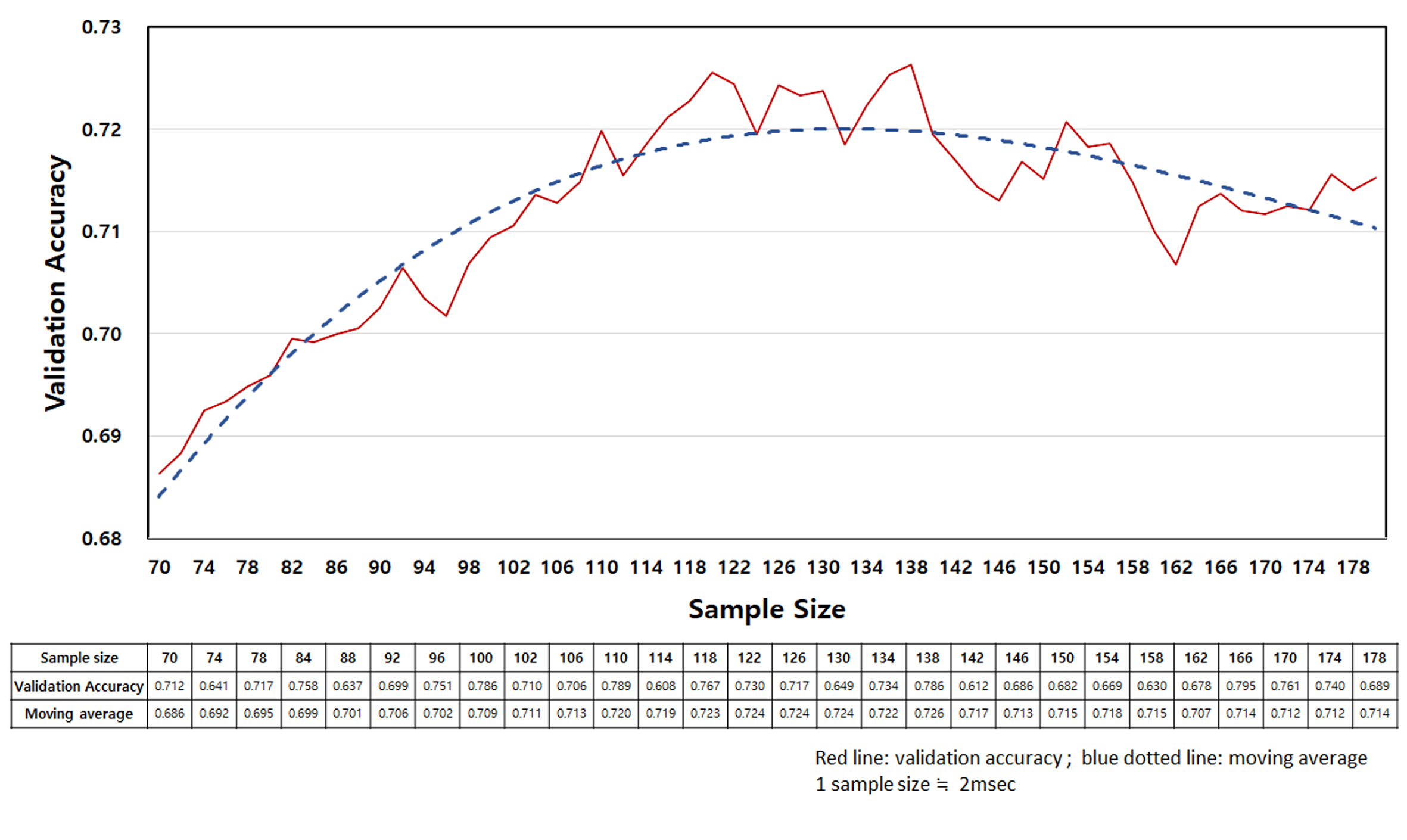

Supplement: Supplementary file 2 — Supplementary Information 1. [file 41598_2021_92172_MOESM2_ESM.tif]
